# Dynamics of postnatal depressive symptoms in early parenthood

**DOI:** 10.1186/s12888-024-05934-6

**Published:** 2024-07-23

**Authors:** Nora Skjerdingstad, Lydia G. Speyer, Adela-Maria Isvoranu, Vibeke Moe, Eivor Fredriksen

**Affiliations:** 1 Department of Psychology, University of Oslo, Oslo, Norway; 2 Department of Psychology, Lancaster University, Lancaster, UK; 3 Department of Psychology, National University of Singapore, Singapore, Singapore

**Keywords:** Postnatal depression, Parental mental health, Graphical Vector Auto-regression, Network analysis

## Abstract

**Background:**

New mothers and fathers are at risk of developing postnatal depressive problems. To understand how postnatal depressive symptoms unfold over time, analyses at the within-person level are necessary. Inspecting postnatal depressive problems at the symptom level provides a novel perspective, ultimately offering insight into which symptoms contribute to the elevation of other symptoms over time.

**Methods:**

Panel graphical vector-autoregression (GVAR) models were applied to analyze the within-person temporal and contemporaneous relations between depressive symptoms across the postnatal period in new mothers and fathers (at T1; *N*_mothers_ = 869, *N*_fathers_ = 579). Depressive symptoms were assessed at 6-, 12-, and 18-months postpartum, using the Edinburgh Postnatal Depression Scale.

**Results:**

The results revealed that for mothers, sadness was a key symptom predicting symptom increases in multiple other depressive symptoms and itself (autoregressive effect) over time. Furthermore, anxiousness and feeling scared predicted each other across the postnatal period in mothers. For fathers, the most central predicting symptom in the overall network of symptoms was being anxious, while self-blame and being overwhelmed had strong self-maintaining roles in the fathers’ symptomatology, indicating that these could be key features in fathers experiencing postnatal depressive problems. The pattern of symptoms that mothers and fathers experienced within the same time window (contemporaneous associations), shared many of the same characteristics compared to the temporal structure.

**Conclusions:**

This study suggests that across the postnatal period, from 6- to 18-months postpartum, depressive symptoms in mothers and fathers contribute differently to the pattern of depressive problems, highlighting sadness as a key feature in maternal symptomatology and anxiousness components in paternal symptomatology.

**Supplementary Information:**

The online version contains supplementary material available at 10.1186/s12888-024-05934-6.

## Introduction

Postnatal depression is a debilitating disorder generating suffering for around 12 to 17% of mothers [1, 2] and between 8 and 13% of fathers [3, 4]. The mental health condition usually occurs during the first year after the child is born and is characterized by a range of depressive symptoms, including sadness and tearfulness, anhedonia, difficulty sleeping, worry, self-blame, and thoughts of self-harm. Postnatal depressive problems tend to be episodic in nature, for some of short duration, while for others, the problems are more persistent. It is recognized that for some parents the disorder can persist well beyond the early postpartum phase, and discerning what characterizes persistent psychopathological patterns of postnatal depressive problems, is warranted [5]. Considering the undesirable impacts of the disorder on the individual parent, family functioning, and child development [6, 7], including later development of child psychopathology and reduced quality of child-mother interactions [8–11], it is imperative to understand more about the growth and persistence of postnatal depressive problems in mothers and fathers. This can ultimately inform preventative efforts aimed at averting the manifestation of the disorder.

Over the past decades, extensive research has been carried out to investigate different features of depressive symptomatology in parents following birth. Several studies have cumulatively led to the understanding of postnatal depression as a heterogeneous condition, in which not only symptom severity, onset, and duration vary among individuals, but also symptom composition [12–14]. Furthermore, research has recognized differences in symptom composition in new mothers versus fathers, with generally lower symptom endorsement in paternal samples and indications of anxiety components being of particular relevance in fathers experiencing postnatal depressive problems [15], underscoring the need to assess these differences in postnatal depressive research.

As the preponderance of studies has been conducted on maternal postnatal depression, researchers have concurrently underscored the lack of knowledge of how and whether paternal symptomatology differs from mothers on a phenomenological level. Studies have highlighted that fathers are not at increased risk for severe depression postpartum, while mothers are [16, 17]. However, prevalence studies which include measures of mild to moderate depression, or use a dimensional approach, show an increased prevalence of postnatal depressive problems in fathers as well. The need to investigate potential differences and similarities between maternal and paternal depressive problems remains imminent [18], particularly since paternal symptomatology is found to be independently associated with negative developmental outcomes in offspring [9]. Maternal depression has been linked to a variety of negative outcomes, such as development of affective disorders in offspring [19], and while the literature on the effect of paternal postnatal depression remains sparse, research suggest that it may have a particular influence on the development of conduct problems in boys [20].

A next step in disentangling depressive problems in new mothers and fathers involves identifying specific symptoms that are sources of the persistence and growth of the symptom load well into the postnatal period. By using methodological and conceptual frameworks that expand our knowledge of how postnatal depressive symptoms unfold over time, this study aims to approach postnatal depressive problems from a network symptom-level perspective. Understanding more about the growth, spread, and continuation of depressive problems at the symptom level is important towards developing a more precise understanding of this broad heterogenous mental health state.

### A Longitudinal Network Approach

Seen through a network theory ‘lens’, activated symptoms can contribute to a mutually interacting system of depressive components, finally reaching the constellation of a full-blown postnatal depressive disorder [21, 22]. Inspecting postnatal depressive problems at the symptom level enables us to gain novel knowledge about symptom patterns that have previously been out of reach. Notably, in line with this reasoning, methodological advances now allow us to investigate how symptom structures change over time, shedding light into potential “driving symptoms” of postnatal depressive problems. This study aims to add to the literature by using a state-of-the-art methodology, specifically the novel panel network approach [23], allowing us to inspect associations between symptoms that occur across and within time.

To further understand the emergence and manifestation of postnatal depressive problems in early parenthood, applications of statistical models that can disaggregate effects that occur at the within- versus between-person level are highly necessary, as denoted by many scholars [24–26]. Within-person effects refer to processes that occur within the individual. For while focusing on mean-level changes in depressive symptoms in early parenthood can give valuable insight into overall trends across individuals, meaningful information about intraindividual variability may be obscured [27]. A recent study highlighted that new parents vary greatly in their within-person experiences of depressive symptoms in the first year postpartum, and that mothers reporting elevated postnatal depressive symptoms at one timepoint were more likely continue to report higher levels at subsequent time points [27], suggesting a carry-over effect over time. Increasing our understanding of the dynamic nature of depressive symptomatology is important to reveal patterns of symptom exacerbation, and one further step is to additionally zoom into which particular symptoms contribute to elevations in other depressive symptoms in this crucial post-partum period.

Despite the known importance of assessing intraindividual changes, empirical investigations of postnatal depressive symptomatology often involve analyses of between-level effects or confound within- and between-person processes. This is particularly problematic as effects occurring on these different levels in some cases can yield opposite patterns. For instance, between-level investigations can shed light into averaged effects between feeling sad and experiencing sleep difficulties (in a population, parents who report more sadness, also report more trouble sleeping), but are limited in grasping whether this association is present temporarily within individuals (whether intraindividual increases in sadness lead to subsequent increases in sleep problems or vice versa). With longitudinal data available for at least three timepoints, recent methodological advances, including the panel network analysis, allow us to separate these effects analytically [26].

The current investigation will describe symptom dynamics contributing to the growth and persistence of postnatal depressive problems. Specifically, we aim to identify symptoms contributing to individual level changes in symptom severity and symptom spread. Moreover, we aim to characterize maternal and paternal patterns of depressive symptom evolution in the postnatal period. Since little research has been conducted longitudinally on the symptom dynamics of postnatal depressive problems, this study will be predominantly exploratory. However, based on existing evidence, we expect that depressive symptoms will predict other symptoms over time [27], and that differences between mothers and fathers will be uncovered. The intraindividual (predicting) associations between the depressive symptoms will be modelled using the Graphical Vector Auto-regression (GVAR) model for panel data [16], and the dataset consists of measurements at 6-, 12-, and 18-months postpartum in new mothers (*N* = 869) and fathers (*N* = 579) taking part in the Little in Norway (LiN) Study.

## Methods

### Study Design and Participants

This study is part of a larger research project, namely the Little in Norway Study (LiN) [28], a cross-disciplinary longitudinal community-based study focusing on child and parental mental health. From September 2011 to October 2012, all pregnant women who received routine prenatal care at nine public well-baby clinics across Norway were invited to participate in the study. The clinics were chosen to ensure diversity in various demographics and were represented from all health regions in Norway. 1041 women initially consented to participate in the study, where five out of these withdrew from the study, leaving a total of 1036 pregnant women. Their partners were also invited to participate, and 884 (878 men and 6 women) agreed to partake. The participants have been followed-up from early pregnancy through infancy and later when the children have entered primary school. This particular study encompasses three data collections in the LiN study consisting of survey data from 6- (T1), 12- (T2) and 18- (T3) months postpartum. Mothers and fathers who responded to a minimum of one of the three timepoints were included in the analyses, and for the purpose of this study, female partners (*n* = 4) were excluded due to the lack of power to produce meaningful conclusions. This was also done as this study’s research questions concern differences between mothers and fathers, and to avoid confounding having given birth with parental gender. In total, 869 mothers and 579 fathers participated at 6 months, 767 mothers (*n* drop-out from T1 = 102, 11.74%) and 551 (*n* drop-out from T1 = 28, 4.84%) fathers at 12 months, and finally 736 mothers (*n* drop-out from T1 = 133, 15.30%) and 519 fathers (*n* drop-out from T1 = 60, 10.36%) enrolled at 18 months postpartum.

Mothers (t (865) = -2.08, *p* = 0.04) and fathers (t (576) = -2.40, *p* = 0.02) that dropped out from T1 to T3 reported significantly higher depressive total scores compared to those participating at T3. No significant differences (*p* > 0.05) were found for age and education comparing mothers at T1 and T3, whereas fathers that participated at T3 had significantly (t (882) = 3.12, *p* = 0.002) higher education than drop-outs.

### Measures

Depressive symptoms were assessed using the Edinburgh Postnatal Scale (EPDS) [29]. The scale was originally developed to deal with the inflation of somatic symptoms that mothers experience in the postpartum period [30], and has later been validated for fathers [15]. The EPDS is a self-report questionnaire consisting of ten items. The respondents are asked to rate various depressive symptoms based on the last seven days on a 4-point Likert Scale. Higher scores on the EPDS indicate more severe depressive problems. In this study, EPDS symptom scores were treated as continuous variables, and each symptom was included as a single item in the analyses. Investigating the distribution of all variables, the item measuring suicidal ideation (EPDS10) was excluded due to a lack of variation. Internal consistency was consistently high for both mothers and fathers across all timepoints, as indicated by Cronbach’s alpha scores ranging from α = 0.84 [0.82, 0.85] at T1, α = 0.81 [0.79, 0.82] at T2, and α = 0.84 [0.83, 86] at T3 for mothers, and α = 0.79 [0.78, 0.81] at T1, α = 0.80 [0.78, 0.81] at T2, and α = 0.78 [0.76, 80] at T3 for fathers. Descriptive statistics for all investigated variables are provided in Table 1 (mothers) and Table 2 (fathers), and the wording of the items is attached in Table S1. The EPDS total scores ranged from 3.02 (*SD* = 3.43) at T1, 2.88 (*SD* = 3.12) at T2, and 3.07 (*SD* = 3.49) at T3 for mothers, while the fathers’ scores ranged from 2.39 (*SD* = 2.89) at T1, 2.20 (*SD* = 2.78) at T2, and 2.09 (*SD* = 2.72) at T3. Furthermore, using a cut-off score of 11 [31] for maternal postpartum depression, 24 mothers (2.77%) scored above clinically significant levels at T2, compared to 19 (2.49%) at T2, and 23 (3.13%) at T3. While studies have suggested cut-off scores ranging from 7–10 [32] for clinically significant postnatal depression in fathers, using a cut-off level of 10 showed that 13 fathers (2.25%) met this level at T1, 9 (1.65%) at T2, and 10 (1.94%) at T3. However, using a cut-off level of 7, indicate that between 4.65% and 6.75% of fathers experience elevated levels of depression across the study period. These rates are generally lower compared to prevalence rates across other paternal [33] and maternal samples [2].

**Table 1:** Descriptive Item Information for Mothers

Items	*N*	*Mean*	*SD*	*Skewness*	*Kurtosis*
6 months postpartum EPDS1	869	.114	.371	3.850	17.948
6 months postpartum EPDS2	869	.119	.379	3.753	16.875
6 months postpartum EPDS3	869	.532	.720	1.177	.662
6 months postpartum EPDS4	869	.667	.786	.790	-.534
6 months postpartum EPDS5	869	.192	.493	2.808	8.213
6 months postpartum EPDS6	869	.648	.681	.640	-.419
6 months postpartum EPDS7	869	.160	.474	3.257	10.949
6 months postpartum EPDS8	867	.405	.611	1.420	1.773
6 months postpartum EPDS9	867	.175	.410	2.203	4.113
6 months postpartum EPDS10	867	.012	.135	15.584	295.739
12 months postpartum EPDS1	767	.098	.342	3.921	17.427
12 months postpartum EPDS2	767	.091	.318	3.900	18.102
12 months postpartum EPDS3	767	.567	.750	1.141	.550
12 months postpartum EPDS4	767	.643	.745	.741	-.635
12 months postpartum EPDS5	767	.173	.454	2.849	8.556
12 months postpartum EPDS6	767	.590	.662	.712	-.446
12 months postpartum EPDS7	767	.163	.464	3.152	10.441
12 months postpartum EPDS8	767	.396	.564	1.162	.850
12 months postpartum EPDS9	765	.134	.363	2.638	6.406
12 months postpartum EPDS10	765	.017	.139	8.880	87.352
18 months postpartum EPDS1	736	.124	.386	3.426	12.682
18 months postpartum EPDS2	736	.110	.358	3.603	14.635
18 months postpartum EPDS3	736	.563	.728	1.057	.288
18 months postpartum EPDS4	736	.641	.762	.855	-.275
18 months postpartum EPDS5	736	.178	.480	2.881	8.241
18 months postpartum EPDS6	736	.671	.708	.613	-.641
18 months postpartum EPDS7	736	.177	.499	3.216	11.056
18 months postpartum EPDS8	735	.415	.584	1.156	.768
18 months postpartum EPDS9	735	.178	.427	2.345	4.929
18 months postpartum EPDS10	735	.016	.137	9.175	93.482

**Table 2:** Descriptive Item Information for Fathers

Items	*N*	*Mean*	*SD*	*Skewness*	*Kurtosis*
6 months postpartum EPDS1	579	.100	.333	3.759	17.108
6 months postpartum EPDS2	579	.123	.382	3.645	16.047
6 months postpartum EPDS3	579	.468	.668	1.251	.867
6 months postpartum EPDS4	579	.473	.691	3.566	.330
6 months postpartum EPDS5	579	.105	.354	2.808	12.906
6 months postpartum EPDS6	579	.565	.689	.881	-.188
6 months postpartum EPDS7	579	.192	.499	2.860	8.517
6 months postpartum EPDS8	578	.304	.534	1.686	2.720
6 months postpartum EPDS9	578	.042	.216	5.599	34.008
6 months postpartum EPDS10	578	.014	.144	11.468	140.959
12 months postpartum EPDS1	551	.089	.327	4.558	26.936
12 months postpartum EPDS2	549	.095	.350	4.468	24.169
12 months postpartum EPDS3	549	.430	.649	1.309	.842
12 months postpartum EPDS4	549	.455	.678	1.219	.315
12 months postpartum EPDS5	549	.080	.278	3.353	10.397
12 months postpartum EPDS6	549	.525	.682	1.002	.047
12 months postpartum EPDS7	547	.144	.431	3.237	10.805
12 months postpartum EPDS8	545	.347	.565	1.453	1.467
12 months postpartum EPDS9	545	.031	.174	5.409	27.352
12 months postpartum EPDS10	545	.011	.121	12.295	167.176
18 months postpartum EPDS1	519	.112	.372	3.978	19.265
18 months postpartum EPDS2	517	.130	.432	4.160	20.266
18 months postpartum EPDS3	517	.383	.613	1.516	1.863
18 months postpartum EPDS4	517	.385	.654	1.666	2.245
18 months postpartum EPDS5	517	.093	.334	3.843	15.218
18 months postpartum EPDS6	516	.494	.664	1.041	.065
18 months postpartum EPDS7	516	.126	.401	3.527	13.408
18 months postpartum EPDS8	516	.331	.544	1.463	1.615
18 months postpartum EPDS9	516	.025	.169	7.249	57.832
18 months postpartum EPDS10	516	.014	.131	10.833	130.392

[Table 1 and 2 about here].

### Statistical Analyses

All statistical procedures were carried out in R (version 4.1.2). Detrending procedures (linear time related effects) were applied to ensure that the data met the assumption of stationarity, implying that the relations between variables are similar between T1 and T2, and between T2 and T3. The variables were also standardized across time points. To handle missing data, full information maximum likelihood (FIML) estimation was employed. FIML produces unbiased estimates provided data are missing at random (MAR) [34].

The study used the multilevel Graphical Vector Auto-regression (GVAR) model for panel data, a method developed by Epskamp [23] and implemented in the *psychonetrics* (version 0.10) package in R [35]. The GVAR model is similar to the Random Intercept Cross Lagged Panel Model (RI-CLPM) [26] within the Structural Equation Modeling (SEM) framework, enabling the investigation of processes that occur within and between individuals. The panel GVAR model uses repeated data (> 3 timepoints) and is well suited to model temporal, contemporaneous, and between-level associations between multiple variables, often visualized as separate network structures [36].

First, the temporal associations between variables are modelled as a combination of a variable’s value and that of all other included variables at the preceding timepoint. In this study, the temporal associations reflect how deviations from within-person means in a depressive symptom are associated with within-person deviations from this mean at the next measurement occasion. Within-person deviation refers to how much an individual differ from how they usually feel. For example, a temporal association between ‘sadness’ and ‘sleep difficulties’ means that ‘sadness’ predicts ‘sleep difficulties’ at the next timepoint (i.e., the time interval applied) while controlling for all other variables. The temporal associations are visualized as a network of variables, where each variable is represented by a node (circle) and the temporal associations are represented by directed edges (arrows) between them. The temporal network can contain associations between separate variables (e.g., between ‘sadness’ and ‘sleep difficulties’ or from and to the same variable (e.g., from ‘sadness’ to ‘sadness’). The latter is referred to as an autoregressive effect or self-loop and implies that a variable predicts itself at the next timepoint. For instance, an autoregressive loop going from ‘sadness’ to ‘sadness’ implies that an on average, an individual who feels sadder than they usually do at one timepoint, will feel increased sadness at the subsequent timepoint. The temporal associations represent effects on the averaged within-person level and are averaged across the 6-months intervals (i.e., not between two specific timepoints).

In addition to the predicting associations discovered in the temporal network, the GVAR model enables us to look at associations between all the investigated variables that occur within the same time window, referred to as contemporaneous effects. The contemporaneous network consists of undirected state-like effects that occur within the same time-window, thus reflecting an overall pattern of symptoms that tend to co-occur, after controlling for the temporal effects. For example, an association between ‘sadness’ and ‘sleep difficulties’ at this level can be interpreted followingly: On average, if an individual is experiencing more sadness than they usually do at one measurement occasion, they also tend to experience more sleep problems over the same time period. The symptoms tend to co-occur. As with the temporal effects, the contemporaneous network reflects averaged within-person associations. In the contemporaneous network, edges between the variables are not directed (lines rather than arrows).

Lastly, to ensure that the model captures within-person effects, between-level effects are controlled for, which refers to mean-level associations across individuals, such as genetic influences, socioeconomic status, or personality traits. The between-level differences are estimated as random intercepts in the model, allowing the temporal and contemporaneous associations to be modelled at the within-person level while simultaneously controlling for these stable trait-like differences.

In sum, the panel GVAR estimation retrieves 1) temporal within-person, 2) contemporaneous within-person, and 3) between-subjects effects, which are visualized as three separate networks (Gaussian Graphical Models) [36] for an intuitive representation of the dependency structures.

The *dvlm1-*function in the *psychometrics* package was used to specify, prune, and select the appropriate models. Pruning and model search procedures were applied to attain sparser network structures, leaving only the most significant edges in the final network. To evaluate fit, Comparative Fit Index (CFI), Tucker Lewis Index (TLI), and Root Mean Square Error of Approximation (RMSEA) were computed, and standard cut off criteria (CFI > 0.90, TLI > 0.90, RMSEA < 0.05) were used to indicate reasonably good fit [37].

Moreover, to quantify the connectedness of each node in the separate networks, centrality metrics were obtained [38]. For the directed temporal network, out-strength and in-strength centrality were estimated, computing the sum of all outgoing (out-strength) and incoming (in-strength) absolute edge weights while excluding the autoregressive effects. Out-strength reveals the extent to which a node predicts other nodes in the network at the next time point, while in-strength displays the extent to which a node is predicted by other nodes at the previous timepoint. For the contemporaneous network, strength centrality is estimated, calculating the sum of all absolute edge weights connected to a node.

Finally, to visualize the networks, the *qgraph* package in R [36] was used. The networks were made comparable by applying the same arrangement of nodes based on the average layout of the within-person networks for mothers. These structures were initially established with the Fruchterman-Reingold algorithm [39] which pushes the most central nodes to the middle and less central nodes to the periphery of the network.

### Stability Analyses

To access the stability of the network structures, 75% of the sample of mothers and fathers respectively was extracted continually as the models were rerun 1000 times. The edges were accordingly coded as either present [1] or not present (0) for each estimated model and the results are displayed as matrices containing the numbers of times each edge is present or not out of 1000 times. For example, if the number is 600, this means that an edge between two specific variables occurred in 60% of the estimated models. If the model did not converge, the edges were counted as not present (0).

## Results

A descriptive summary of the sample is provided in Table 3. The saturated GVAR models including all potential edges showed good fit for both mothers (TLI = 0.98, CFI = 0.99 and RMSEA = 0.024) and acceptable fit for fathers (TLI = 0.89, CFI = 0.93 and RMSEA = 0.049), while the pruned models performed slightly better than the saturated models for mothers (∆BIC = 464.52). For fathers the saturated model performed better than the pruned model (∆BIC = 135.66), potentially indicating that the associations between variables are better captured in the saturated model. However, the sparser pruned network is included for interpretation and comparative purposes, but these results need to be interpreted with caution. All model fit statistics are provided in Supplementary Table S4.

**Table 3:** Demographic Characteristics of the Participants

	**Mothers** (*N* = 869)	**Fathers** (*N* = 579)
**Age**	32.29 (5.73)	32.32 (5.56)
**Education level** Primary school level High school level University level (< 4 years) University level (> 4 years) Not specified	21 (2.4%) 167 (19.2%) 329 (37.9%) 352 (40.5%) -	20 (3.5%) 151 (26.1%) 184 (31.8%) 216 (37.3%) 8 (1.4%)
**Employment status** Full-time employment Full-time or part-time student Part-time employed Student and employed Unemployed / on benefits / homemakers Not specified	676 (77.8%) 50 (5.8%) 63 (7.2%) 52 (6.0%) 21 (2.4%) 7 (.8%)	511 (88.3%) 21 (3.6%) 7 (1.2%) 24 (4.1%) 5 (0.9%) 11 (1.9%)
**Civil status** Married Cohabitant Single / separated Not specified	308 (35.4%) 531 (61.1%) 22 (2.5%) 8 (.9%)	209 (36.1%) 355 (61.3%) 7 (1.2%) 12 (2.1%)
**Ethnicity** Norwegian Other Not specified	825 (94.9%) 44 (5.1%) -	552 (95.3%) 19 (3.3%) 8 (1.4%)
**Number of children prior to birth** 0 1 2 3 or more Not specified	473 (54.4%) 292 (33.6%) 91 (10.5%) 13 (1.5%) -	328 (56.6%) 187 (32.3%) 45 (7.8%) 11 (1.9%) 8
**First pregnancy** Yes No	347 (39.9%) 522 (60.1%)	- -

**Fig. 1:**
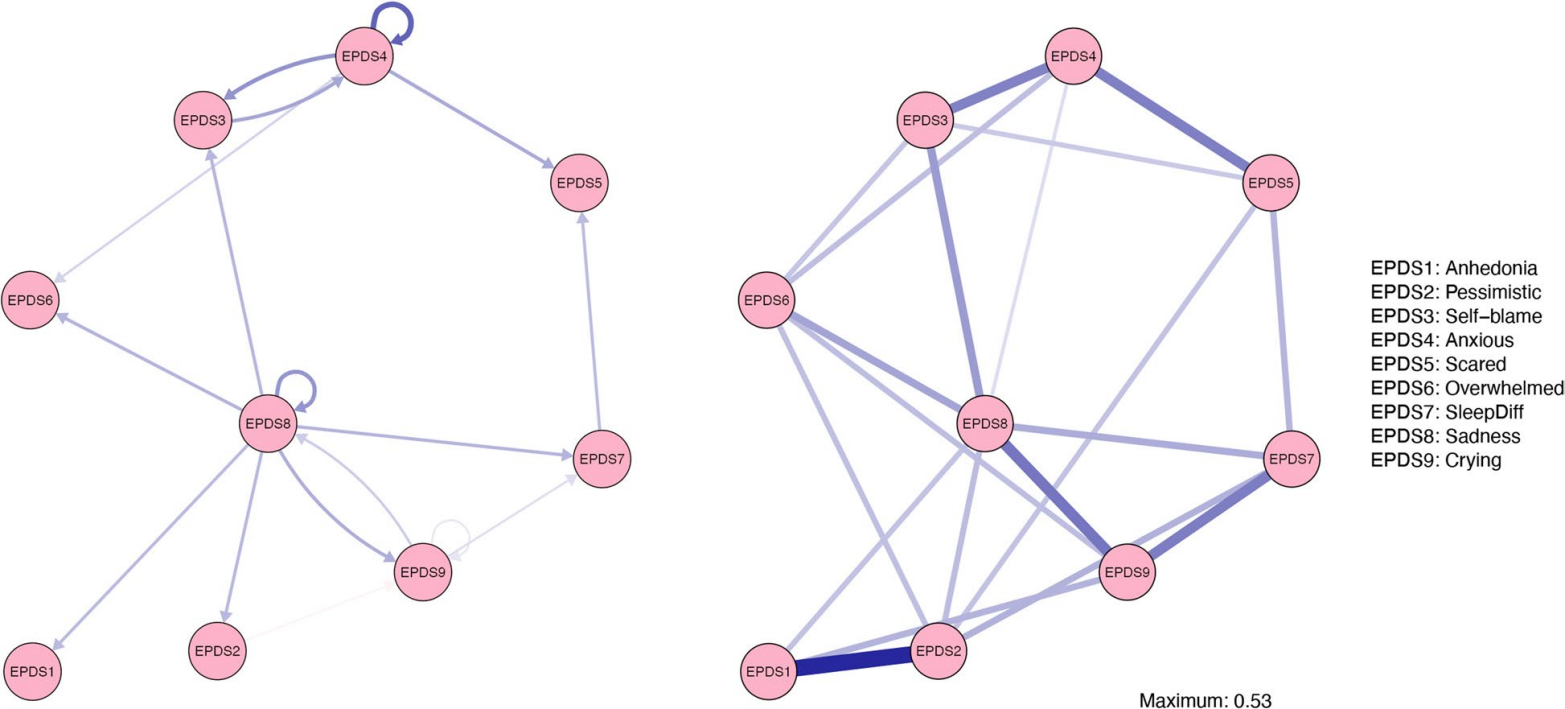
Mothers’ Temporal Network (left panel) and Contemporaneous Network (right panel). Note. Each node (circle) represents a postnatal depressive symptom

For mothers, the pruned temporal and contemporaneous networks are presented in Fig. 1. Each node in the network represent a postnatal depressive symptom based on the EPDS scale. The temporal network (left panel in Fig. 1) contains information about predicting relations between the depressive symptoms over time, as depicted by arrows going from and to different nodes, whereas the contemporaneous network (right panel in Fig. 1) reflects associations occurring within the same time window. How central a symptom is in the overall network structure (i.e., how well a node predicts and is predicted by other nodes), captured by strength estimates for the temporal and contemporaneous effects, is presented in Table 4. The specific edge weight estimates for both network structures are attached to Supplementary Table S5 and S6.

**Table 4:** Centrality Estimates for Each Variable in Mothers’ Temporal and Contemporaneous Network

	**Temporal Network**		**Contemporaneous Network**
**Node name**	**Instrength**	**Outstrength**	**Strength Centrality**
EPDS1 Anhedonia	.14	.00	.73
EPDS2 Pessimistic	.15	.02	1.00
EPDS3 Self-blame	.37	.18	.69
EPDS4 Anxious	.18	.49	.73
EPDS5 Scared	.31	.00	.65
EPDS6 Overwhelmed	.24	.00	.70
EPDS7 SleepDiff	.22	.14	.75
EPDS8 Sadness	.10	.91	1.18
EPDS9 Crying	.19	.17	.84

In the maternal sample, sadness displayed the most predicting associations to other symptoms in the temporal network structure. From sadness at one timepoint several directed edges were present; to sleep difficulties, crying, anhedonia, pessimistic, self-blame, and overwhelmed, highlighting sadness as a key feature increasing the spread of postnatal depressive symptoms. Additionally, sadness displayed a strong autoregressive effect, indicating that mothers who feel sad at one timepoint also experience more sadness six months later after controlling for all other variables in the network. A bidirectional loop was further present between sadness and crying, where more crying predicted more sadness at the next timepoint and vice versa, suggesting a reinforcing loop over time between these symptoms.

Anxiousness displayed the second highest out-strength (Table 4) following sadness in the mothers’ temporal network. Anxiousness predicted self-blame, feeling overwhelmed, and being scared in six months intervals. Anxiousness also showed a strong autoregressive effect, and a bidirectional loop with self-blame was also present, suggesting that anxiousness and self-blame reinforce each other over time. The temporal network for mothers thus revealed that sadness and anxiousness were the most central symptoms in predicting an increase in depressive symptom spread and severity at six months intervals in the postnatal period.

Self-blame was the most predicted symptom (highest in-strength; Table 4), but overall, the estimates were generally evenly distributed compared to the out-strength estimates. On the other hand, anhedonia, scared, overwhelmed and pessimistic, did not display any strong outgoing effects or autoregressive effects, suggesting that these symptoms are less important in the maintenance of depressive problems in mothers across the postnatal period.

The contemporaneous network shows associations between variables that unfold within the same time window (Fig. 1, right panel). Sadness was revealed to have the highest strength centrality (Table 4), with positive relations to all other postnatal depressive symptoms except being scared. The strongest edge weights were present between pessimistic and anhedonia, between crying and sadness, and between crying and sleep difficulties.

**Fig. 2:**
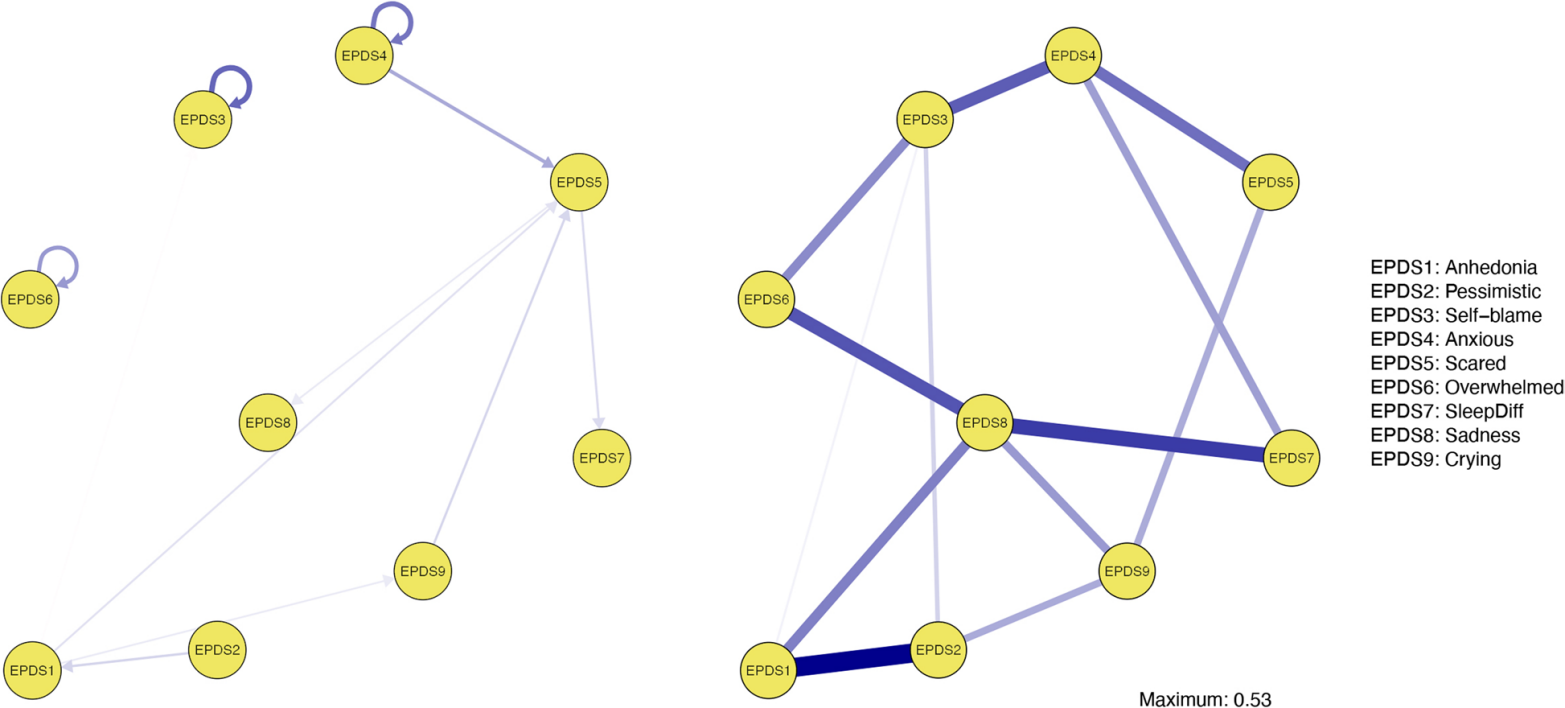
Fathers’ Temporal Network (left panel) and Contemporaneous Network (right panel). Note. The arrangements of nodes are based on the average of the mothers’ temporal and contemporaneous networks for comparison

The networks containing information about the structure of paternal postnatal depressive symptoms are presented in Fig. 2, with edge weights presented in Supplementary Figure S7 and Figure S8. The interpretation of the results for fathers should be done with more caution as the fathers’ networks were computed with fewer individuals (lower power). The strongest directed edge occurred between being anxious at one timepoint and being more scared at the next timepoint. Additionally, strong autoregressive effects occurred for self-blame, anxiousness, and feeling overwhelmed, reflecting how these variables predict themselves at consecutive timepoints for fathers. Inspecting the out-strength and in-strength estimates (Table 5), anxiousness had the overall strongest predictive effect on all other symptoms followed by anhedonia and pessimistic, whereas scared was the variable most prone to being influenced by other variables across time. The contemporaneous effects for the fathers are visualized in Fig. 3 (right panel), and the strength centrality estimates for each variable are presented in Table 5. Sadness was contemporaneously and positively associated with overwhelmed, sleep difficulties, crying, and anhedonia.

**Table 5:** Centrality Estimates for Each Variable in Fathers’ Temporal and Contemporaneous Network

	**Temporal Network**		**Contemporaneous Network**
**Node name**	**Instrength**	**Outstrength**	**Strength Centrality**
EPDS1 Anhedonia	.09	.11	.83
EPDS2 Pessimistic	.00	.09	.80
EPDS3 Self-blame	.004	.00	.70
EPDS4 Anxious	.00	.18	.84
EPDS5 Scared	.32	.12	.48
EPDS6 Overwhelmed	.00	.00	.60
EPDS7 SleepDiff	.07	.00	.61
EPDS8 Sadness	.04	.00	1.25
EPDS9 Crying	.04	.08	.56

Comparing the paternal and maternal postnatal symptom patterns, several differences emerged. For mothers, sadness and being anxious had the most directed edges towards other symptoms. For the fathers, anxiousness and being scared were revealed to have the highest out-strength, whereas sadness (the highest out-strength for mothers) had no temporal connections to other symptoms. Three autoregressive temporal effects were present in the fathers’ network, namely for anxiousness, self-blame, and feeling overwhelmed, while for mothers’ autoregressive loops were revealed for anxiousness and sadness. Followingly, anxiousness predicting itself at the next timepoint was common across mothers and fathers, whereas sadness was especially important for mothers, and self-blame and being overwhelmed for fathers. However, as a part of a bidirectional loop with anxiousness, self-blame was also part of overall pattern in mothers.

### Network Stability

The results from the stability analyses are presented in Table S9 – S12 in Supplementary materials and the tables contain information about how many out of the 1000 replications of the model edges were present or not. Generally, the temporal networks were less stable compared to the contemporaneous networks, which could suggest that the networks may be less sparse than indicated by the pruned networks. Particularly, one edge that was displayed in the mothers’ temporal network (from sadness to pessimistic) was below 10% of the 1000 replications and should be interpreted with caution. All temporal edges present in the pruned network for fathers were displayed more than 36% of the 1000 replications of the model.

## Discussion

The main purpose of the current study was to identify specific symptoms contributing to the growth and maintenance of postnatal depressive problems in mothers and fathers. Investigating how these symptoms unfold within individuals is crucial to understand the formation and maintenance of the symptomatology in the postnatal period.

A panel network approach was applied to unveil associations between postnatal depressive symptoms in mothers and fathers with the aim of understanding their mental health state on a more granular level. This study goes beyond similar cross-sectional findings [40] by providing longitudinal information about the role of sadness in reinforcing other symptoms over time. Sadness was identified as the most influential symptom across time in mothers, indicating that mothers who feel more sadness in the postnatal period, also experience increases in sadness and multiple other depressive symptoms (i.e., crying, overwhelmed, self-blame, difficulties sleeping) at consecutive timepoints. As such, the symptom can act as an amplifier in the depressive symptom structure in mothers, also indicated by being the node with highest out-strength centrality, ultimately suggesting that maternal sadness should be addressed at an early stage after birth to potentially avoid the constellation of postnatal depressive problems.

Being anxious seems to play an important role as an amplifier across time in mothers and fathers. Mothers reporting being more anxious than their own average (more than usual), tend to report subsequent increases in self-blame and vice versa. More within-person anxiousness was also associated with feeling more scared and overwhelmed, in addition to feeling more anxious six months later. For fathers, anxiousness was also found to be an important component of depressive problems over time, in. A population-based study on Swedish parents resonates well with this finding as researchers found that the EPDS seems to pick up more distress rather than pure depression in new fathers [41]. Other symptoms that are not captured by the measure, including irritability, helplessness, and frustration could be more prominent in paternal depressive symptomatology [42]. High comorbidity of anxiety and depression in the postnatal period for both mothers and fathers has been reported in previous studies [43, 44].

Additionally, a cross-sectional study [40] found that being scared was the most central symptom in fathers, and this study extends this finding by pointing out that over time, this symptom is rather on the receiving end (highest in-strength) than contributing to amplification of symptoms over time. This underlines the necessity to complement cross-sectional findings with studies including repeated measures that can extract directed associations on the within-person level.

Furthermore, the passive features of the maternal depressive symptomatology (i.e., anhedonia and pessimistic) together with feeling scared and feeling overwhelmed showed no temporal influence on other symptoms over time, suggesting that these symptoms do not contribute to elevations of depressive symptomatology across six months intervals in mothers. This is somewhat surprising considering that anhedonia and inactivity are often linked to and seen as maintaining factors of depression. As this study uses data from a community sample with few mean EPDS scores above cut-off (> 11), these results might be different in a clinical sample with higher levels of depression and anhedonia scores in particular. It should also be mentioned that the lack of edges could result from lower network stability, as demonstrated by the conducted stability analyses showing lower stability for the temporal associations. Furthermore, the findings should be interpreted in light of the measurement intervals applied in this study. For while this study uncovered averaged temporal associations between symptoms across months, the exact temporal nature of each symptom remains unclear and might be better captured by other time intervals (e.g., across days or weeks). Thus, future investigations should investigate the dynamic nature of depressive symptoms across different time scales. Accumulatively, more efforts are needed to capture and investigate the development of depressive symptoms in parents.

The study further investigated how postnatal symptoms were connected within the same time window, reflecting which symptoms tend to co-occur in mothers and fathers. Here, the associations between symptoms are more similar across mothers and fathers compared the pattern discovered in the temporal analyses. Sadness was revealed to be the symptom with most within-person associations to other variables in both groups. Thus, mothers and fathers who feel more sadness than their own average, also tend to experience a range of other postnatal depressive symptoms within the same timeframe. This suggest that feeling sad is a prominent symptom in both mothers and fathers, which is often accompanied by a range of other postnatal depressive symptoms.

Overall, the results indicate that the contemporaneous symptom profiles in new mothers and fathers seem to be quite similar, while the symptoms contributing to the elevation of postnatal depressive symptoms over time are revealed to differ across mother and fathers. These preliminary results are important to consider in the assessment, treatment, and future research on the disorder. An important aspect is that fathers and mothers who experience elevated depression following birth, may require different treatment and prevention efforts. Specifically, the study highlights what contributes to elevation and manifestation of depressive symptomatology beyond the first six months postpartum. Elevations in sadness in this period may be particularly relevant to track and detect in mothers, while anxiousness seems to be more prominent in paternal symptomatology over the same period.

### Limitations and Future Studies

This study should be considered in light of several imitations. One limitation is the representativity of the sample, with the preponderance of both mothers and fathers reporting high education levels, full-time employment, and having a partner. Future studies should investigate the generalizability of the results in more representative samples. Another limitation is that higher levels of depression were associated with attrition in this study, suggesting that mothers who are more severely depressed tend to discontinue the study over time. This could potentially produce bias in the results, limiting the generalizability of the findings to more depressed mothers. To address this, additional sensitivity analyses were conducted using only participants with complete data (See Supplementary Figure S1 and S2). The results from these analyses for mothers showed that the edges from the original analyses were replicated, but that fewer edges emerged. Additionally, the model showed poorer fit (CFI/TLI = 0.92, RMSEA = 0.052). This is likely due to the loss of power by not using the full sample. However, this should be noted as a limitation of the study. For fathers, the networks containing only complete cases revealed to be less stable, as reflected by poor fit metrics (CFI/TLI = 0.67/0.68, RMSEA = 0.096). Only a few edges appeared in the temporal network, and these results should be interpreted as unreliable due to low power. Another related limitation is that while we conducted stability analyses to assess whether the results replicated across multiple subsets of the data, the temporal pattern of depressive symptoms was generally more unstable compared to the relations within the same time frame. We highlight the importance of conducting future replications of this study in larger samples.

Another limitation is that the study is limited to the understanding of postnatal depressive symptoms in a period ranging from 6 to 18 months postpartum. Moreover, while this study aims to provide information about the differences in postnatal symptom patterns in mothers and fathers across the postnatal period, no formal significance testing between the two groups were performed due to lack of such procedures within this novel panel network framework. Next, due to the lack of variation in the measure of suicidal ideation, this item was excluded from further analyses, making it impossible to draw any conclusions about the role of this variable in postnatal symptomatology. The item endorsement was furthermore less variable for fathers compared to the mothers, possibly reflecting that fathers may experience other symptoms in the postnatal period that are not captured by the measure (EPDS) used in this study. The sample of fathers furthermore contained fewer participants, possibly leading to some estimation problems. Despite this, the comparison of mothers and fathers is often left out in the literature because of non-respondent fathers. This study considers the benefits of including fathers as larger than the limitations tied to it.

The study further treats a 4-point Likert scale as continuous. Recent simulations [45] have demonstrated that the use of skewed ordinal data is valid for network estimation, but future studies should uncover how ordinal data affects panel network models specifically. Additionally, the results from the stability analyses show that the temporal networks are less stable compared to the contemporaneous networks. Some edges in the mothers’ temporal network were particularly unstable, and this should be considered an important limitation in the study. Finally, the analyses were exploratory in nature, and it is highly necessary that the findings are replicated in independent data in addition to using more confirmatory modelling strategies.

However, the application of a novel method, despite its limitations, also contributes to addressing postnatal depressive problems from a new perspective. In addition, contrasting cross-sectional investigations, this study applies a model that enables the crucial disaggregation of within- and between-subject effects.

## Conclusion

This study adds to the literature by examining how networks of depressive symptoms unfold in the postnatal period for mothers and fathers. The results suggest that experiencing more sadness at the within-person level reinforces most other depressive symptoms over time in mothers, while self-blame and anxiousness mutually reinforce each other in the postnatal period. Fathers’ temporal network showed that anxiousness was the most influential symptom over time, displaying a strong self-reinforcing effect over the postnatal period. The resulting symptom patterns within the same timeframe indicated that both new mothers and fathers tend to experience similar co-occurring depressive symptoms. By examining the dynamics of postnatal depressive problems from a symptom level perspective, this study provides a starting point for future inspections of the disorder.

### Supplementary Information

Supplementary Material 1.

## Data Availability

The data that support the findings of this study is not publicly available due to restrictions applied to the dataset. However, the data can be made available upon reasonable request by e-mailing P.I. VM (co-author).
