# Supplementary material for: Dynamics of postnatal depressive symptoms in early parenthood
Source: BMC Psychiatry. 2024 Jul 23;24:523. doi: 10.1186/s12888-024-05934-6 (PMC11264399; doi:10.1186/s12888-024-05934-6)
Supplement: Supplementary file 1 — Supplementary Material 1. [file 12888_2024_5934_MOESM1_ESM.docx]

**Supplementary Materials**

| **Table S3**  *Item Wordings (12 items)* | | |
| --- | --- | --- |
| **No.** | **Node Name** | **Item** |
| EPDS1 | Anhedonia | I have been able to laugh and see the funny side of things. |
| EPDS2 | Pessimistic | I have looked forward with enjoyment to things |
| EPDS3 | Self-blame | I have blamed myself unnecessarily when things went wrong |
| EPDS4 | Anxious | I have been anxious or worried for no good reason |
| EPDS5 | Scared | I have felt scared or panicky for no very good reason |
| EPDS6 | Overwhelmed | Things have been getting on top of me |
| EPDS7 | Sleep difficulties | I have been so unhappy that I have had difficulty sleeping. |
| EPDS8 | Sadness | I have felt sad or miserable. |
| EPDS9 | Crying | I have been so unhappy that I have been crying. |
| *Note.* All items were measured on a four-point scale (1-4) | | |

| **Table S4**  *Model Fit Statistics* | | | | | | |
| --- | --- | --- | --- | --- | --- | --- |
|  |  | *X^2^* | RMSEA | TLI/CFI | AIC | BIC |
| **Mothers** | Saturated Model | 343.68 | .024 | .98/.99 | 52102.51 | 52967.34 |
|  | Prune + stepup | 641.28 | .032 | .96/.96 | 52176.11 | 52502.82 |
| **Fathers** | Saturated Model | 600.87 | .049 | .89/.93 | 37315.60 | 38135.31 |
|  | Prune+ stepup | 1581.99 | .070 | .76/.76 | 38038.72 | 38270.97 |

**Table S5**

*Edge Weights – Temporal Network Mothers (Pruned)*

|  | EPDS1 | EPDS2 | EPDS3 | EPDS4 | EPDS5 | EPDS6 | EPDS7 | EPDS8 | EPDS9 |
| --- | --- | --- | --- | --- | --- | --- | --- | --- | --- |
| EPDS1 | _ | _ | _ | _ | _ | _ | _ | _ | _ |
| EPDS2 | _ | _ | _ | _ | _ | _ | _ | _ | -.022 |
| EPDS3 | _ | _ | _ | .183 | _ | _ | _ | _ | _ |
| EPDS4 | _ | _ | .221 | .324 | .175 | .089 | _ | _ | _ |
| EPDS5 | _ | _ | _ | _ | _ | _ | _ | _ | _ |
| EPDS6 | _ | _ | _ | _ | _ | _ | _ | _ | _ |
| EPDS7 | _ | _ | _ | _ | .138 | _ | _ | _ | _ |
| EPDS8 | .140 | .145 | .151 | _ | _ | .154 | .153 | .218 | .167 |
| EPDS9 | _ | _ | _ | _ | _ | _ | .069 | .104 | .050 |

**Table S6**

*Edge Weights – Contemporaneous Network Mothers (Pruned)*

|  | EPDS1 | EPDS2 | EPDS3 | EPDS4 | EPDS5 | EPDS6 | EPDS7 | EPDS8 | EPDS9 |
| --- | --- | --- | --- | --- | --- | --- | --- | --- | --- |
| EPDS1 |  |  |  |  |  |  |  |  |  |
| EPDS2 | 0.446 |  |  |  |  |  |  |  |  |
| EPDS3 | _ | _ |  |  |  |  |  |  |  |
| EPDS4 | _ | _ | .260 |  |  |  |  |  |  |
| EPDS5 | _ | .125 | .110 | .263 |  |  |  |  |  |
| EPDS6 | _ | .132 | .118 | .133 | _ |  |  |  |  |
| EPDS7 | _ | .160 | _ | _ | .151 | _ |  |  |  |
| EPDS8 | .125 | .138 | .205 | .069 | _ | .186 | .170 |  |  |
| EPDS9 | .156 | _ | _ | _ | _ | .134 | .268 | .285 |  |

**Table S7**

*Edge Weights – Temporal Network Fathers (Pruned)*

|  | EPDS1 | EPDS2 | EPDS3 | EPDS4 | EPDS5 | EPDS6 | EPDS7 | EPDS8 | EPDS9 |
| --- | --- | --- | --- | --- | --- | --- | --- | --- | --- |
| EPDS1 | _ | _ | -.004 | _ | .060 | _ | _ | _ | .041 |
| EPDS2 | .087 | _ | _ | _ | _ | _ | _ | _ | _ |
| EPDS3 | _ | _ | .318 | _ | _ | _ | _ | _ | _ |
| EPDS4 | _ | _ | _ | .287 | .177 | _ | _ | _ | _ |
| EPDS5 | _ | _ | _ | _ | _ | _ | .074 | .044 | _ |
| EPDS6 | _ | _ | _ | _ | _ | .212 | _ | _ | _ |
| EPDS7 | _ | _ | _ | _ | _ | _ | _ | _ | _ |
| EPDS8 | _ | _ | _ | _ | _ | _ | _ | _ | _ |
| EPDS9 | _ | _ | _ | _ | .083 | _ | _ | _ | _ |

**Table S8**

*Edge Weights – Contemporaneous Network Fathers (Pruned)*

|  | EPDS1 | EPDS2 | EPDS3 | EPDS4 | EPDS5 | EPDS6 | EPDS7 | EPDS8 | EPDS9 |
| --- | --- | --- | --- | --- | --- | --- | --- | --- | --- |
| EPDS1 |  |  |  |  |  |  |  |  |  |
| EPDS2 | .535 |  |  |  |  |  |  |  |  |
| EPDS3 | .024 | .093 |  |  |  |  |  |  |  |
| EPDS4 | _ | _ | .340 |  |  |  |  |  |  |
| EPDS5 | _ | _ | _ | .301 |  |  |  |  |  |
| EPDS6 | _ | _ | .243 | _ | _ |  |  |  |  |
| EPDS7 | _ | _ | _ | .202 | _ | _ |  |  |  |
| EPDS8 | .266 | _ | _ | _ | _ | .358 | .411 |  |  |
| EPDS9 | _ | .176 | _ | _ | .174 | _ | _ | .212 |  |

**Table S9**

*Stability Analysis for Mothers’ Temporal Network – 1000 bootstraps*

|  | EPDS1 | EPDS2 | EPDS3 | EPDS4 | EPDS5 | EPDS6 | EPDS7 | EPDS8 | EPDS9 |
| --- | --- | --- | --- | --- | --- | --- | --- | --- | --- |
| EPDS1 | 413 | 403 | 51 | 94 | 88 | 89 | 64 | 245 | 199 |
| EPDS2 | 277 | 347 | 217 | 31 | 90 | 64 | 214 | 204 | 482 |
| EPDS3 | 195 | 174 | 380 | 695 | 339 | 289 | 311 | 172 | 273 |
| EPDS4 | 45 | 61 | 682 | 796 | 550 | 460 | 136 | 281 | 119 |
| EPDS5 | 116 | 279 | 132 | 108 | 340 | 184 | 119 | 71 | 88 |
| EPDS6 | 71 | 50 | 129 | 93 | 152 | 403 | 29 | 126 | 45 |
| EPDS7 | 120 | 308 | 166 | 103 | 559 | 50 | 429 | 434 | 210 |
| EPDS8 | 106 | 73 | 259 | 65 | 211 | 288 | 278 | 529 | 311 |
| EPDS9 | 95 | 355 | 90 | 32 | 85 | 48 | 150 | 395 | 302 |

**Table S10**

*Stability Analysis for Mothers’ Contemporaneous Network – 1000 bootstraps*

|  | EPDS1 | EPDS2 | EPDS3 | EPDS4 | EPDS5 | EPDS6 | EPDS7 | EPDS8 | EPDS9 |
| --- | --- | --- | --- | --- | --- | --- | --- | --- | --- |
| EPDS1 |  |  |  |  |  |  |  |  |  |
| EPDS2 | 976 |  |  |  |  |  |  |  |  |
| EPDS3 | 58 | 77 |  |  |  |  |  |  |  |
| EPDS4 | 180 | 72 | 926 |  |  |  |  |  |  |
| EPDS5 | 81 | 802 | 696 | 975 |  |  |  |  |  |
| EPDS6 | 339 | 587 | 623 | 623 | 236 |  |  |  |  |
| EPDS7 | 131 | 855 | 297 | 61 | 643 | 131 |  |  |  |
| EPDS8 | 819 | 732 | 949 | 451 | 138 | 951 | 959 |  |  |
| EPDS9 | 847 | 258 | 249 | 41 | 220 | 699 | 971 | 976 |  |

**Table S11**

*Stability Analysis for Fathers’ Temporal Network – 1000 bootstraps*

|  | EPDS1 | EPDS2 | EPDS3 | EPDS4 | EPDS5 | EPDS6 | EPDS7 | EPDS8 | EPDS9 |
| --- | --- | --- | --- | --- | --- | --- | --- | --- | --- |
| EPDS1 | 618 | 569 | 368 | 182 | 555 | 190 | 340 | 236 | 537 |
| EPDS2 | 679 | 557 | 243 | 176 | 403 | 65 | 315 | 175 | 431 |
| EPDS3 | 113 | 148 | 776 | 264 | 184 | 413 | 95 | 335 | 173 |
| EPDS4 | 186 | 225 | 474 | 716 | 600 | 396 | 162 | 255 | 360 |
| EPDS5 | 109 | 261 | 422 | 582 | 706 | 174 | 419 | 552 | 338 |
| EPDS6 | 129 | 104 | 343 | 138 | 163 | 666 | 197 | 455 | 298 |
| EPDS7 | 220 | 228 | 156 | 184 | 212 | 252 | 607 | 137 | 304 |
| EPDS8 | 202 | 206 | 140 | 215 | 196 | 648 | 259 | 619 | 247 |
| EPDS9 | 310 | 212 | 90 | 183 | 471 | 165 | 362 | 228 | 659 |

**Table S12**

*Stability Analysis for Fathers’ Contemporaneous Network – 1000 bootstraps*

|  | EPDS1 | EPDS2 | EPDS3 | EPDS4 | EPDS5 | EPDS6 | EPDS7 | EPDS8 | EPDS9 |
| --- | --- | --- | --- | --- | --- | --- | --- | --- | --- |
| EPDS1 |  |  |  |  |  |  |  |  |  |
| EPDS2 | 1000 |  |  |  |  |  |  |  |  |
| EPDS3 | 355 | 366 |  |  |  |  |  |  |  |
| EPDS4 | 143 | 289 | 946 |  |  |  |  |  |  |
| EPDS5 | 159 | 279 | 457 | 752 |  |  |  |  |  |
| EPDS6 | 298 | 650 | 706 | 600 | 211 |  |  |  |  |
| EPDS7 | 249 | 530 | 407 | 528 | 619 | 398 |  |  |  |
| EPDS8 | 918 | 455 | 575 | 810 | 231 | 989 | 988 |  |  |
| EPDS9 | 499 | 745 | 142 | 84 | 729 | 173 | 507 | 764 |  |

**Fig. S1**

*Mothers’ Temporal Network (left panel) and Contemporaneous Network (right panel) Conducted with Only Complete Cases (*n *= 652)*

*Note.* This model had poorer fit compared to the original analyses; CFI/TLI = .92, RMSEA =.052

**Fig. S2**

*Fathers’ Temporal Network (left panel) and Contemporaneous Network (right panel) Conducted with Only Complete Cases (*n *= 379)*

*Note.* The fit of this model proved to be low (CFI/TLI = .67/.68, RMSEA = .096)
